# Would *Zika virus* Infection in Pregnancy Be a Sentence of Poor Neurological Prognosis for Exposed Children? Neurodevelopmental Outcomes in a Cohort from Brazilian Amazon

**DOI:** 10.3390/v14122659

**Published:** 2022-11-28

**Authors:** Marília Rosa Abtibol-Bernardino, Lucíola de Fátima Albuquerque de Almeida Peixoto, Marcia da Costa Castilho, Camila Helena Aguiar Bôtto-Menezes, Silvana Gomes Benzecry, Rodrigo Haruo Otani, Gabriela Ribeiro Ivo Rodrigues, Beatriz Caroline Soares Chaves, Geruza Alfaia de Oliveira, Cristina de Souza Rodrigues, Flor Ernestina Martinez-Espinosa, Maria das Graças Costa Alecrim

**Affiliations:** 1 Postgraduate Program in Tropical Medicine (PPGMT), State University of Amazonas (UEA) in Partnership with the Tropical Medicine Foundation Dr Heitor Vieira Dourado (FMT-HVD), Manaus 69040-000, Brazil; 2 Department of Maternal and Child Health, Medical School, Federal University of Amazonas (UFAM), Manaus 69020-160, Brazil; 3 Department of Virology, Tropical Medicine Foundation Dr Heitor Vieira Dourado (FMT-HVD), Manaus 69040-000, Brazil; 4 Department of Medicine, School of Health Sciences, State University of Amazonas (UEA), Manaus 69065-001, Brazil; 5 Department of Malaria, Tropical Medicine Foundation Dr Heitor Vieira Dourado (FMT-HVD), Manaus 69040-000, Brazil; 6 Postgraduate Program in Living Conditions and Health Situations in the Amazon (PPGVIDA), Leônidas & Maria Deane Institute at Fiocruz Amazonia, Manaus 69057-070, Brazil; 7 Laboratory of Territory Environment Health and Sustainability, Leônidas & Maria Deane Institute of Fiocruz Amazonia, Manaus 69057-070, Brazil; 8 Medical Course Coordination at Manaus Metropolitan College/FAMETRO, Manaus 69050-000, Brazil

**Keywords:** arbovirus, *Zika virus*, children, congenital *Zika virus* syndrome, coinfection, neurodevelopment, neurologic manifestations, non-microcephalic children, microcephaly

## Abstract

Infections with Flavivirus in pregnant women are not associated with vertical transmission. However, in 2015, severe cases of congenital infection were reported during the *Zika virus* outbreak in Brazil. More subtle infections in children born to mothers with ZIKV still remain uncertain and the spectrum of this new congenital syndrome is still under construction. This study describes outcomes regarding neurodevelopment and neurological examination in the first years of life, of a cohort of 77 children born to pregnant women with ZIKV infection in Manaus, Brazil, from 2017 to 2020. In the group of normocephalic children (92.2%), most showed satisfactory performance in neuropsychomotor development, with a delay in 29.6% and changes in neurological examination in 27.1%, with two children showing muscle-strength deficits. All microcephalic children (5.2%) evolved with severe neuropsychomotor-development delay, spastic tetraparesis, and alterations in the imaging exam. In this cohort, 10.5% of the children had macrocephaly at birth, but only 2.6% remained in this classification. Although microcephaly has been considered as the main marker of congenital-*Zika-virus* syndrome in previous studies, its absence does not exclude the possibility of the syndrome. This highlights the importance of clinical follow-up, regardless of the classification of head circumference at birth.

## 1. Introduction

Infections by Flavivirus in pregnant women are generally not associated with vertical transmission, nor do they cause severe changes in the developing fetus. In 2015, severe cases of congenital infection were reported during the *Zika virus* (ZIKV) epidemic in Brazil, which led to the issue of an international alert by the World Health Organization (WHO) [1,2,3].

In the city of Manaus, the capital of the Amazonas state, located in the northern region of Brazil, the year 2015 marked the beginning of the ZIKV epidemic. According to the 68th Epidemiological Report released in January 2017 by the Municipal Department of Health, 6123 cases of *ZIKV* fever have been registered in the capital, since the beginning of official notifications in 2015. Of this total, 4418 cases were confirmed, 1682 were discarded, and 23 were still under investigation. About 1286 cases of ZIKV infection were reported in pregnant women, with 500 cases confirmed, 773 discarded, and 13 under investigation. Regarding microcephaly, the document presents 38 notifications. Of this total, five cases of microcephaly related to ZIKV were confirmed, 11 were discarded, and 11 others were still under investigation. There were also 11 confirmed cases of microcephaly not related to ZIKV [4].

Experimental models have demonstrated that the Brazilian strain of ZIKV is able to cross the placental barrier, infect progenitor cells, and induce apoptosis and autophagy, leading to cell death, reduction of neuronal-proliferative zones, and disruption in the organization of cerebral-cortical layers [5]. Congenital *Zika virus* syndrome (CZVS) has such main markers as microcephaly, intracranial calcifications, malformations of cortical development, reduced volume of white and gray brain matter, and ventriculomegaly.

Although the cases with severe involvement are already well defined, the less severe cases of children who do not present stigmata at birth still remain uncertain [6,7,8,9]. Therefore, the phenotypic spectrum of this syndrome is still under construction. This study intends to contribute by describing the neurological outcomes related to intrauterine exposure to ZIKV at any gestational moment, in the first years of life, in a cohort of children from Manaus, Brazil.

## 2. Materials and Methods

### 2.1. Study Design

This is an observational, longitudinal, and prospective study of neurological outcomes related to neuropsychomotor development and physical examination of a cohort of children who suffered exposure to ZIKV in the intrauterine period.

### 2.2. Study Population

The children were born in the period from March 2016 to June 2018, from mothers with laboratory-confirmed infections by this ZIKV at any time of gestation. This is part of the project, “Epidemiological, clinical, nutrological, virological, histopathological and immunological characteristics of ZIKV infection in pregnant women with acute exanthematous disease and its relationship with microcephaly or possible adverse outcomes in Manaus, Amazonas” [10]. Parents or legal guardians agreeing to the participation of their children signed an informed consent form.

### 2.3. Patient Care

In the period from March 2017 to August 2020, children were followed by a multidisciplinary team and underwent at least one evaluation with a neuropediatrician. Prenatal and birth-history data was collected from interviews with the mothers, from information on the pregnant woman’s and child’s medical cards, and from the FMT-HVD electronic-medical record. A multidisciplinary team consisting of a pediatrician, pediatric neurologist, and pediatric nutrologist performed a general and specialized clinical evaluation of the children. Birth anthropometry was analyzed according to sex and gestational age at birth, based on the charts standardized by the International Fetal and Newborn Growth Consortium for the 21st Century (INTERGROWTH-21st), using the software, Neonatal Size Calculator, for newborn infants between 24 + 0 and 42 + 6 gestation weeks (v1.0.6257.25111; The Global Health Network/ University of Oxford, OX, UK) [11,12]. In the case of prematurity, chronological age was corrected during the first two years of life, according to Babson [13]. During follow-up, the remaining anthropometric measurements were based on the WHO standardization, WHO Child Growth Standards, through the WHO Anthro Software (v3.2.2; WHO, GE, CH), an instrument created to monitor growth in the children’s population from 0 to 60 months [14,15].

Neuropsychomotor development was evaluated using the Child Development Surveillance Instrument contained in the child’s medical card, recommended by the Brazilian Health Ministry [16,17]. In some children, it was possible to apply the Bayley-III Scales of Infant and Toddler Development (Pearson, London, UK), already described in a series of cases [18]. Children with suspected developmental delay were thoroughly evaluated by the neuropediatrician from the team to confirm or exclude such triaged deficit.

The neurological examination was performed by the neuropediatrician on all children, following Prechtl’s principle of minimal manipulation [19]. The child’s interaction, communicative and social intentions, crying characteristics, posture and attitude, general mobility, muscle strength pattern, presence of abnormal tone, characteristics of osteotendinous reflexes (bicipital, triceps, radial, patellar, and achilles), and cranial nerve (CN) alterations were evaluated. Using a measuring tape, Head Circumference (HC) was measured considering the largest occipito-frontal diameter, and craniofacial proportion, cranial symmetry, fontanelles, and sutures were also verified. The reflexes (R.) of Moro, asymmetrical tonic-cervical R., palmar grasp R., plantar grasp R., stepping R., Babikin, and Gallant’s R. were evaluated. The Landau reflex evaluated the extensor tonus of the trunk and neck, helping in the differential diagnosis between infant’s pathological or physiological hypotonia. [20].

The criteria for indicating imaging tests, such as cranial tomography (CT) and/or brain magnetic resonance imaging (MRI), were: detection of abnormalities in HC at birth; developmental delay; or abnormalities in neurological examination over the follow-up period.

### 2.4. Laboratory Testing

ZIKV infection was confirmed through detection by Real-Time Reverse Transcriptase Polymerase Chain Reaction (RT-PCR) technique in blood or urine samples from the pregnant women with exanthematous syndrome. The serum samples were collected within the first five days of symptom onset, while the collection of urine samples was within the first eight days. The examination was performed following the protocol of Lanciotti et al. at the Central Laboratory of Public Health (LACEN) in the same city [21]. Tests for Dengue-virus and Parvovirus-B19-virus infections were performed by the Virology Laboratory at the FMT-HVD, using Dengue Virus IgM Capture and Parvovirus B19 IgM kits (DxSelectTM Focus Diagnostics, Cypress, CA, USA). The detection of etiological agents of TORCH Syndrome was mainly performed at the Clinical Analysis Laboratory of the FMT-HVD; however, new tests were not performed, if the patient had recent test results from another (public or private network) laboratory. Malaria was investigated through a thick blood smear test in the FMT-HVD, but, only in pregnant women with a positive clinical-epidemiological history.

### 2.5. Definitions

The presence of at least one of the following findings was considered in the definition of CZVS: microcephaly with partial collapse of the skull, thinning of the cerebral cortex with subcortical calcifications, congenital contractures, presence of spastic hypertonia, and signs of extrapyramidal involvement [22].

Microcephaly was considered when the HC Z score was lower than −2 standard deviations (SDs) and was classified as severe in the occurrence of values lower than −3 SDs. Macrocephaly was considered when HC values were greater than +2 SDs, and severe, when these were greater than +3 SDs.

The occurrence of other outcomes, such as epilepsy, autistic spectrum disorder (ASD), and cerebral palsy were also evaluated. Epilepsy is defined as the occurrence of two or more unprovoked seizures separated by intervals of more than 24 h, or one unprovoked seizure but with a probability of new seizures similar to the overall risk of recurrence (at least 60%) after two unprovoked seizures [23]. The diagnosis of ASD was based on the criteria established by the American Psychiatric Association contained in the Diagnostic and Statistical Manual of Mental Disorders 5th edition (DSM−5). The basic principle is the presence of persistent impairment in communicative and social intention, associated with repetitive and stereotyped patterns of behavior and interests [24]. Cerebral Palsy is a chronic, non-progressive encephalopathy characterized by permanent neurological changes affecting motor development, with impairment on movement and body posture. The motor-deficit patterns were characterized by tetraparesis (muscle- strength deficit in the four limbs), paraparesis (strength deficit predominantly in the lower limbs), and hemiparesis (impairment of one half of the body), and may be present with a spastic and/or dystonic pattern, associated with osteotendinous hyperreflexia. In order to classify the degree of motor dysfunction in daily life, Gross Motor Function Classification System (GMFCS) was used in 5 levels for cerebral palsy [25].

### 2.6. Statistical Analysis

The Stata 14 statistical software package was used for data analysis. Categorical variables were expressed as absolute frequencies. Continuous variables were expressed as average and standard deviations. For statistical purposes, the children were divided into 3 groups according to the classification of HC: microcephalic, normocephalic, and macrocephalic. To test the association between two nominal variables, the Fisher’s exact test was used.

## 3. Results

In 2016, 828 pregnant women with exanthematous syndrome were followed at the Tropical Medicine Foundation Dr Heitor Vieira Dourado (FMT-HVD), of which 328 (39.6%) received laboratory confirmations of a ZIKV infection. After delivery, 78 women, representing 23.78% of those with a confirmed infection, continued in a follow-up program, allowing pediatric follow-up of their children. One of these women was excluded from the present study because she had not received a neurological evaluation.

The average of neurological assessments of the 77 children followed in this cohort was 2.6 consultations (SD ± 1.25), with ages ranging between 2 and 51 months, and a median age, at the last assessment, of 33 months. Table 1 shows the sociodemographic and prenatal clinical characteristics of the 77 pregnant women, according to the classification of head circumference of the newborn at birth.

**Figure 1:**
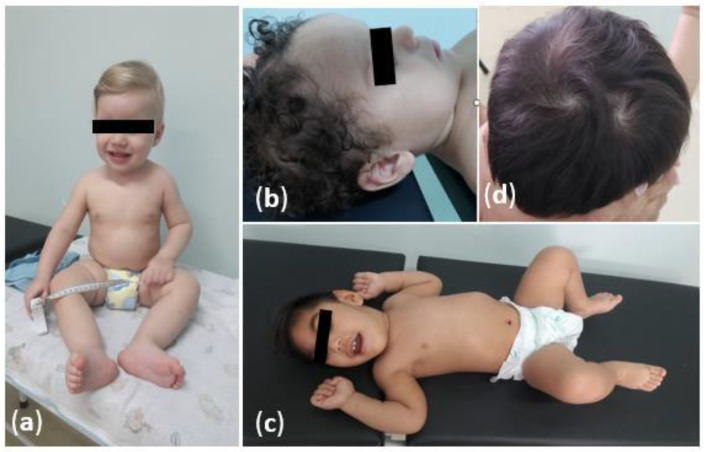
Physical characteristics of children exposed to *Zika virus* in the intrauterine period, in Manaus, Brazil: (**a**) A normocephalic boy without neuropsychomotor-developmental delays or abnormalities in the physical-neurological examination; (**b**) A child with macrocephaly which evolved to normalization of the cephalic perimeter; (**c**) A boy with microcephaly and spastic tetraparesis due to congenital-*Zika-virus* syndrome; (**d**) Supernumerary-hair-swirl alteration.

At least one dysmorphic change was detected in 15/77 (19.5%) children. Of these, 4/15 (26.6%) were microcephalic, 10/15 (66.7%) were normocephalic, and 1/15 (10%) were macrocephalic. All microcephalic patients had craniofacial disproportion, bitemporal depression, excess skin on the nape and scalp, prominent occipital bulge, epicanthus, retrognathia, and strabismus. Among the normocephalic patients, 10/65 (15.4%) dysmorphic findings were craniofacial disproportion (1/10, 10%), supernumerary-capillary swirl (1/10, 10%), high palate (1/10, 10%), hemangioma (1/10, 10%), iris-color change (1/10, 10%), familial syndactyly (1/10, 10%), cutis agenesis (1/10, 10%), epicanthus (2 /10, 20%), flat nasal base (2/10, 20%), and hyperchromic spots (2/10, 20%). The macrocephalic child at birth (1/8, 12.5%) presented a supernumerary-hair-swirl alteration. Figure 1 shows physical characteristics of children followed in the Manaus cohort, Brazil. Table 2 describes the characteristics of birth and neonatal period according to the classification of the children’s head circumference.

As for the classification of head circumference at birth, all four children with microcephaly were considered proportional to their lengths, and only one (25%) child was classified as having a severe form. However, during the clinical follow-up, all evolved to the severe form of microcephaly. None of this subgroup has been diagnosed with co-infection with another pathogen. Among the eight children with macrocephaly at birth, four (50%) were proportional to their height and six (75%) evolved to normocephaly in the first years of life. In the main group, 6/77 (7.8%) children were diagnosed with ZVCS (four microcephalic and two normocephalic). One child (1/77, 1.3%) was diagnosed with a genetic-neurosensory hearing loss due to a mutation in the *GJB2* gene. Table 3 summarizes the neurological outcomes related to neuropsychomotor development and physical examination after the follow-up period of the 77 exposed children, and Table 4 shows the distribution of outcomes according to HC.

Table 5 shows the findings of imaging exams (CT and/or MRI) of the children with alterations in head circumference, developmental delay, or alterations in the neurological-physical examination. The most severe image abnormalities were present in the group of children with spastic tetraparesis, comprising all microcephalic and one normocephalic children, all with exposure in the first gestational trimester. The second normocephalic child, who presented hemiparesis suggestive of CZVS, did not show changes to the image. Two other normocephalic children with hemiparesis were premature, and presented delayed neuropsychomotor development, abnormalities in neurological and in imaging exams (periventricular leukomalacia) secondary to this condition. The only macrocephalic patient with image alteration was diagnosed with autism.

In this cohort, 19.5% of the children were exposed to other agents in the intrauterine period. Those coinfected with Epstein-Barr virus, hepatitis B, Dengue, and Plasmodium vivax did not show adverse neurological outcomes. Of the five (6.5%) children infected with the herpes-simplex virus, 2.6% had a mild delay in cognitive and language development. Among the two (2.6%) coinfected with Parvovirus B19, 1.2% presented language delay. Of the two (2.6%) children diagnosed with *Toxoplasma gondii*, one (1.2%) was diagnosed with autism. The children with the most significant abnormalities were those exposed to HIV during pregnancy, in which, one showed scaphocephaly and motor-development delay, and the other, macrocephaly and developed epilepsy in the third year of life. No child with changes in the imaging exam had coinfection.

## 4. Discussion

The fact that *Zika virus* has been revealed as a new teratogenic pathogen with a devastating neurotropism in the developing fetal brain has motivated clinical observation of exposed children during pregnancy [2,3,7]. Cases marked by the presence of microcephaly have been widely documented in the literature, but neurological outcomes of children born without stigmas of the congenital syndrome remain uncertain [10,26]. The present cohort includes the outcomes of 77 children exposed to ZIKV during pregnancy, stratified according to a head-circumference classification, and with the vast majority being non-microcephalic children.

The severity of fetal involvement following exposure to an infectious agent depends on the susceptibility of fetal genotype to the teratogenic agent, how it interacts with various environmental factors, and how it varies with the degree of embryonic development at the time of exposure [27]. The children with the most severe involvement were those with congenital microcephaly, all of whom had been exposed to ZIKV in the first gestational trimester and were not coinfected by other pathogens. They evolved with marked retardation in neuropsychomotor development and clinical features compatible with cerebral palsy classified, according to GMFCS, at level V, with spastic tetraparesis, tendon hyperreflexia, and cranial nerve alterations. The images showed alterations such as intracranial calcifications, dilatation of the ventricular system, lissencephaly, and cortico-subcortical atrophy, similar to cases of CZVS described by other cohorts [8,9,26]. Lissencephaly present in the neuroimaging of all four children with microcephaly is a malformation of cortical development secondary to a neuronal-migration disorder that can lead to severe development delay, intellectual disability, and epilepsy refractory to drug treatment [28,29]. This malformation would justify the fact that all children in this microcephalic subgroup have epilepsy.

The evolution of exposed and asymopmatic children at birth have been a gap in spectrum description of ZIKV involvement. Efforts have been made to construct a scenario of intrauterine-exposure involvement by reporting on several national and international cohorts [9,30,31,32,33,34,35,36,37]. In this study, it was observed that most children with normocephaly performed well in the evaluation of neuropsychomotor development, with an occurrence of delay in approximately one-third of the children, with language being the domain with the greatest impairment. In the neurological examination, 72.9% had no alteration.

Despite the satisfactory results in children without HC alterations, one child evolved with severe neurological impairment, with spastic tetraparesis classified as GMFCS level V, severe delay in neuropsychomotor development, epilepsy, and pachygyria on MRI. This malformation is part of the lissencephaly complex, which is also responsible for the clinical manifestations described above, fulfilling criteria for CZVS, despite the normal cranial circumference. Another normocephalic child had clinical but milder signs of cerebral palsy with GMFCS level I, manifested as spastic hemiparesis that became evident after the first year of life, with no other risk factors besides intrauterine exposure to ZIKV. Two other children had hemiparesis, but the cause was justified by prematurity, with imaging findings compatible with this event (periventricular leukomalacia). These cases may corroborate the information that, despite microcephaly being the main marker of CZVS, its absence does not exclude the possibility of the occurrence of the syndrome [34,38]. This emphasizes the importance of clinical follow-up of all exposed children, regardless of the classification of head circumference at birth. Although there is evidence that abnormalities can occur at any gestational period, studies carried out in several countries have shown that fetal-ZIKV infections that occur in late gestational stages are most often associated with subtle manifestations at birth, while those that occur in early stages usually present more severe manifestations, as seen in this cohort [35,36,37,39].

An unusual finding was that 10.4% of children had macrocephaly at birth, but none was severe. However, at the end of the follow-up period, 75% had their HC normalized, migrating to the normocephaly group. The occipitofrontal circumference reflects the brain volume, but can also be influenced by other factors, such as cranial thickness, cerebrospinal-fluid collections, and ventricular volume [40]. Mild cases of macrocephaly can occur in individuals without neurological alterations, which justifies the cases that had their head circumference normalized without clinical and neuroimaging alterations [41]. Among those who maintained macrocephaly, one received a diagnosis of autism with an image showing megacisterna magna and the other, with exposure associated with HIV, presented transfontanellar ultrasound with mild, third-ventricle dilation in the second month of life; nonetheless, in the third year, when epilepsy started, brain-magnetic-resonance imaging showed no abnormalities. Studies have suggested the pathophysiological possibility of intrauterine exposure to ZIKVpredisposing the occurrence of autism [42]. However, the association of macrocephaly and autism, which has also been described, generally related to genetic causes of this disorder, and therefore, was less likely to be related to intrauterine infection by ZIKV [43].

The congenital infection by *ZIKV*, alone, already has a catastrophic potential. It is also important to know the behavior of this exposure associated with other pathogens in pregnancy. The children in this cohort, coinfected by *Epstein Barr virus*, *Hepatitis B virus*, *Dengue virus*, and *Plasmodium vivax*, showed no abnormalities related to head circumference, neuropsychomotor development, or neurological examination. On the other hand, the two exposed to HIV had neuropsychomotor development delay, and one had congenital macrocephaly maintained until the end of the follow-up period associated with epilepsy. It is known that isolated exposure to HIV, even in uninfected children, may trigger an immune reaction leading to the production of a cascade of cytokines that may interfere with neuronal migration and axonal growth, and, by itself, causing damage to the child’s neurological development [44,45]. Activation of the maternal-inflammatory process, enteric-environmental dysfunction, and antiretroviral toxicity are also risk factors for impaired neurodevelopment in these children [46]. Children exposed to herpes, parvovirus B19, and toxoplasmosis also showed developmental delay.

One of the limitations of this study is the difficulty to adhere to the follow-up visits by the pregnant women’s families. It was because the exposed children were asymptomatic at birth, and the families believed that their children were free from risk. Because it was a long follow-up period, the families had difficulty in maintaining the regular frequency of evaluations, and it was not possible to standardize the visits at the same age. Outdated registration data posed another obstacle to recruiting research participants and keeping them in follow-up. It was not possible to add a group that was certainly not exposed to ZIKV during pregnancy in order to compare the outcomes between the exposed and unexposed. This is because, considering the possibility of occurrence of asymptomatic- maternal cases, every child born in the epidemic period was at risk of having been exposed to ZIKV, despite the absence of symptoms during pregnancy. Another fact is due to the high number of dengue cases in our region, which can generate cross-immunological response in ZIKV-serological tests, making false results possible. Studies with accurate comparative groups that safely exclude the possibility of asymptomatic exposure to the virus during pregnancy still need to be conducted.

## 5. Conclusions

Despite the potential to cause severe neurological impairment in children exposed in the intrauterine period, the diagnosis of *Zika-virus* infection during pregnancy was not a sentence of poor prognosis for all children in this cohort from the Brazilian Amazon. The composition of this cohort was mostly normocephalic children, who showed satisfactory performance in neurodevelopment throughout the first year of life. Although the most severe neurological findings have been described in symptomatic children with microcephaly at birth, cases of CZVS have also been described in the non-microcephalic group. This emphasizes the importance of multidisciplinary-clinical follow-up in exposed children, regardless of the head-circumference classification at birth. Campaigns to promote the eradication of proliferative-mosquito foci and the use of repellents in pregnant women should be encouraged to prevent this threatening congenital infection.

## Figures and Tables

**Table 1:** Sociodemographic and clinical characteristics of 77 pregnant women with positive RT-PCR for *Zika virus*, distributed according to head circumference at childbirth, Manaus, Brazil.

Baseline Characteristics	Microcephaly n/Total	Normocephaly n/Total	Macrocephaly n/Total	n/Total (%)
Age (average ± SD)	22.5 ± 6.6	28 ± 6.4	27.7 ± 6.3	27.8 ± 6.3
≤19 years, n(%)	0/4 (0%)	6/65 (9.3%)	0/8 (0%)	6/77 (7.8%)
20–34 years, n(%)	4/4 (100%)	47/65 (72.1%)	6/8 (75%)	57/77 (74%)
≥35 years, n(%)	0/4 (0%)	12/65 (18.6%)	2/8 (25%)	14/77 (18.2%)
Years of schooling				
0–8, n(%)	2/3 (66.7%)	8/62 (12.9%)	2/8 (25%)	12/73 (16.5%)
9–11, n(%)	1/3 (33.3%)	36/62 (58.1%)	2/8 (25%)	39/73 (53.4%)
>12 years, n(%)	0/3 (0%)	18/62 (29%)	4/8 (50%)	22/73 (30.1%)
Hypertensive disease, n(%)	1/4 (25%)	10/65 (15.4%)	0/8 (0%)	11/77 (14.3%)
Gestational diabetes, n(%)	0/4 (0%)	1/65 (1.5%)	2/8 (25%)	3/77 (3.9%)
Tobacco intake, n(%)	0/4 (0%)	1/65 (1.5%)	0/8 (0%)	1/77 (1.3%)
Alcohol intake, n(%)	0/4 (0%)	2/65 (3%)	0/8 (0%)	2/77 (2.6%)
Illicit drugs intake, n(%)	0/4 (0%)	0/65 (0%)	0/8 (0%)	0/77 (0%)
Gestational bleeding, n(%)	0/4 (0%)	3/65 (4.6%)	1/8 (12.5%)	4/77 (5.2%)
Depression, n(%)	0/4 (0%)	1/65 (1.5%)	0/8 (0%)	1/77 (1.3%)
Trimester of ZIKV infection				
1st trimester, n(%)	4/4 (100%)	13/65 (20.1%)	2/8 (25%)	19/77 (24.6%)
2nd trimester, n(%)	0/4 (0%)	24/65 (36.9%)	5/8 (62.5%)	29/77 (37.7%)
3rd trimester, n(%)	0/4 (0%)	28/65 (43%)	1/8 (12.5%)	29/77 (37.7%)
Coinfection occurrence, n (%)	0/4 (0%)	13/65 (20.1%) *	2/8 (25%) *	15/77 (19.5%) *
Herpes simplex type 1 and 2, n(%)	0/4 (0%)	4/65 (6.1%)	1/8 (12.5%)	5/77 (6.5%)
Parvovirus B19, n(%)	0/4 (0%)	1/65 (1.5%)	1/8 (12.5%)	2/77 (2.6%)
HIV, n(%)	0/4 (0%)	1/65 (1.5%)	1/8 (12.5%)	2/77 (2.6%)
Epistein-Barr, n(%)	0/4 (0%)	1/65 (1.5%)	0/8 (0%)	1/77 (1.3%)
Hepatitis B, n(%)	0/4 (0%)	1/65 (1.5%)	0/8 (0%)	1/77 (1.3%)
Dengue, n(%)	0/4 (0%)	4/65 (6.1%)	0/8 (0%)	4/77 (5.2%)
Toxoplasmosis, n(%)	0/4 (0%)	2/65 (3%)	0/8 (0%)	2/77 (2.6%)
Malaria, n(%)	0/4 (0%)	1/65 (1.5%)	0/8 (0%)	1/77 (1.3%)

* Some pregnant women have been coinfected with more than one infectious agent

**Table 2:** Characteristics related to birth and neonatal period observed in 77 children exposed to *Zika virus* in the intrauterine period distributed according to the head circumference at birth, Manaus, Brazil.

Birth and Neonatal Variables	Microcephaly n/Total	Normocephaly n/Total	Macrocephaly n/Total	Total
Type of delivery				
Vaginal	1/4 (25%)	33/65 (50.8%)	1/8 (12.5%)	35/77 (45.5%)
C-section	3/4 (75 %)	32/65 (49.2%)	7/8 (87.5%)	42/77 (54.5%)
Gender				
Male	3/4 (75 %)	26/65 (40%)	7/8 (87.5%)	36/77 (46.7%)
Female	1/4 (25%)	39/65 (60%)	1/8 (12.5%)	41/77 (53.3%)
Apgar at 5th min < 7	0/4 (0%)	1/64 (1.6%)	0/7 (0%)	1/75 (1.3%)
Gestational age, (average ± SD)	39.2 ± 0.9	39 ± 0.9	39 ± 1	39 ± 0.96
Prematurity	0/4 (0%)	5/65 (7.7%)	0/8 (0%)	5/77 (6.5%)
Intracranial bleeding				
Grades I and II	0/4 (0%)	3/65 (4.6%)	0/8 (0%)	3/77 (3.9 %)
Grades III and IV	1/4 (25%)	0/65 (0%)	0/8 (0%)	1/77 (1.3%)
Neonatal sepsis	0/4 (0%)	5/65 (7.7%)	0/8 (0%)	5/77 (6.5%)
Neonatal jaundice	1/4 (25%)	15/65 (23%)	2/8 (25%)	18/77 (23.4%)
Neonatal crises	3/4 (75 %)	0/65 (0%)	0/8 (0%)	3/77 (3.9 %)
Hyaline membrane disease	0/4 (0%)	1/65 (1.5%)	0/8 (0%)	1/77 (1.3%)
Pulmonary Bronchodysplasia	0/4 (0%)	1/65 (1.5%)	0/8 (0%)	1/77 (1.3%)

**Table 3:** Neurological outcomes related to neuropsychomotor development and physical examination at the end of the follow-up period of 77 children exposed to *Zika virus* in the intrauterine period, Manaus, Brazil.

Neurological Findings	N (%)	IC (95%)
Head circumference		
Normocephaly	71 (92.2%)	83.5–96.5
Microcephaly	4 (5.2%)	1.9–13.3
Macrocephaly	2 (2.6%)	0.6–10.1
Neurologic examination with at least one abnormality	25 (32.5%)	22.8–43.9
Hemiparesis *	3 (3.9%)	1.2–11.7
Tetraparesis	5 (6.5%)	2.7–14.9
Hypotonia	1 (1.3%)	0.2–9.0
Spastic hypertonia	7 (9.1%)	4.3–18.1
Osteotendinous hyperreflexia	9 (11.7%)	6.1–21.2
Cranial nerves abnormalities	7 (9.1%)	4.3–18.1
Neuropsychomotor development	26 (33.8%)	23.9–45.2
Cognitive skills	9 (11.7%)	6.1–21.2
Language skills	23 (29.9%)	20.5–41.2
Motor skills	14 (18.2%)	10.9–28.7
Psychosocial skills	11 (14.3%)	8.0–24.2
Autism	4 (5.2%)	1.9–13.3
Epilepsy	6 (7.8%)	3.5–16.5
Dysphagia	7 (9.1%)	4.3–18.1

* Two children with hemiparesis secondary to prematurity.

**Table 4:** Neurological findings related to neuropsychomotor development and physical examination according to head circumference classified at the end of the follow-up period of 77 children exposed to the *Zika virus* in the intrauterine period, Manaus, Brazil.

Neurological Findings	Normocephaly (n = 71)	Microcephaly (n = 4)	Macrocephaly (n = 2)	*p*-Valor
Neurologic examination with at least one abnormality	19 (26.7%)	4 (100%)	2 (100%)	0.001
Deficits in social interaction	6 (8.4%)	4 (100%)	0 (0%)	0.001
Deficit of muscle strength	4 (5.6%)	4 (100%)	0 (0%)	<0.001
Muscle-tonus abnormalities	4 (5.6%)	4 (100%)	0 (0%)	<0.001
Osteotendinous Hyperreflexia	5 (7.0%)	4 (100%)	0 (0%)	<0.001
Cranial-nerves abnormalities	3 (4.2%)	4 (100%)	0 (0%)	<0.001
Epilepsy	1 (1.4%)	4 (100%)	1 (50%)	<0.001
Neurodevelopment delay	21 (29.6%)	4 (100%)	1 (50%)	0.006
Cognitive skills	5 (7.0%)	4 (100%)	0 (0%)	<0.001
Language skills	18 (25.3%)	4 (100%)	1 (50%)	0.022
Motor skills	9 (12.7%)	4 (100%)	1 (50%)	<0.001
Psychosocial skills	7 (9.9%)	4 (100%)	0 (0%)	<0.001
Dysmorphisms	9 (12.7%)	4 (100%)	1 (50%)	<0.001
Autism	4 (5.6%)	0 (0%)	0 (0%)	1.000

**Table 5:** Neuroimaging findings according to the classification of head circumference at birth of 29 children exposed to *Zika virus* in the intrauterine period, Manaus, Brazil.

Neuroimaging Features	Microcephaly (n = 4)	Normocephaly (n = 20)	Macrocephaly (n = 5)	n/Total (%) (n = 29)	IC (95%)
Abnormal Neuroimaging	4/4 (100%)	3/20 (15%)	1/5 (20%)	8/29 (27.6%)	13.8–47.5
Cerebral calcifications	4/4 (100%)	1/20 (5%)	0/5 (0%)	5/29 (17.2%)	7.0–37.7
Ventricular dilatation	4/4 (100%)	1/20 (5%)	0/5 (0%)	6/29 (20.7%)	9.1–40.4
Lissencephaly/ pachygyria	4/4 (100%)	1/20 (5%)	0/5 (0%)	5/29 (17.2%)	7.0–37.7
Cortico-subcortical atrophy	4/4 (100%)	2/20 (10%)	0/5 (0%)	6/29 (20.7%)	9.1–40.4
Megacisterna magna	0/4 (0%)	0/20 (0%)	1/5 (20%)	1/29 (3.4%)	0.4–23.0
Periventricular leukomalacia	0/4 (0%)	2/20 (10%)	0/5 (0%)	2/29 (6.9%)	1.6–25.4
